# Network meta-analysis of first-line immune checkpoint inhibitor therapy in advanced non-squamous non-small cell lung cancer patients with PD-L1 expression ≥ 50%

**DOI:** 10.1186/s12885-023-11285-4

**Published:** 2023-08-23

**Authors:** Wei Chen, Jiayi Chen, Lin Zhang, Sheng Cheng, Junxian Yu

**Affiliations:** 1 Department of Pharmacy, Beijing Friendship Hospital, Capital Medical University, Beijing, China; 2 School of Nursing, Capital Medical University, Beijing, China

**Keywords:** Non-small cell lung cancer (NSCLC), Immune checkpoint inhibitors (ICIs), Programmed cell death-1 (PD-1), Programmed cell death ligand-1 (PD-L1), Network meta-analysis (NMA)

## Abstract

**Introduction:**

The optimal first-line immunotherapy regimen for advanced non-squamous non-small cell lung cancer (NS-NSCLC) patients with programmed cell death ligand 1 (PD-L1) expression ≥ 50% remains unclear. Our aim is to determine the most effective treatment regimen through a network meta-analysis (NMA) comparing these treatments.

**Methods:**

A systematic search was performed in PubMed, Cochrane Library, Web of Science, and Embase databases, and a Bayesian network meta-analysis was conducted. To ensure transparency, the study was registered in the International Prospective Register of Systematic Reviews (CRD42022349712).

**Results:**

The analysis included 11 randomized controlled trials (RCTs) with 2037 patients and 12 immunotherapy combinations. ICI-ICI, ICI alone, and chemotherapy-ICI showed significant advantages over chemotherapy in terms of overall survival (OS) and progression-free survival (PFS). Pembrolizumab plus chemotherapy showed the best OS results compared to chemotherapy. Tislelizumab plus chemotherapy and sintilimab plus chemotherapy provided the best PFS results.

**Conclusions:**

For NS-NSCLC patients with PD-L1 ≥ 50%, pembrolizumab plus chemotherapy, tislelizumab plus chemotherapy, and sintilimab plus chemotherapy are recommended as good treatment options based on the results of this Network meta-analysis (NMA).

## Introduction

Lung cancer has the highest mortality rate of all cancers and is the second most common cancer, after female breast cancer[1]. NSCLC accounts for approximately 85% of all lung cancers, with NS-NSCLC comprising more than half of all NSCLC cases[2, 3]. Molecular targeted therapy is the standard first-line treatment for patients with advanced NSCLC with sensitive genetic mutations. In contrast, platinum-based dual chemotherapy is the standard first-line treatment for patients without targeted gene alterations or with unknown mutation status[4, 5]. While conventional chemotherapy is the mainstay of treatment for advanced NSCLC, its clinical benefits are limited. It has a median OS of less than one year and a five-year PFS rate of only 4%[6, 7]. However, the emergence of immune checkpoint inhibitors (ICIs) offers new hope for patients with advanced NSCLC.

ICIs work by activating the anti-tumor activity of T-lymphocytes through the inhibition of the interaction between PD-1, PD-L1, and cytotoxic T lymphocyte-associated antigen-4 (CTLA-4). This interaction removes tumor cells and tumor tissue to achieve anti-tumor effects[8, 9]. Additionally, ICIs primarily target cancer antigens and prevent normal cells from being attacked [10]. In recent years, ICIs have been approved for use in advanced NSCLC and have been widely used in clinical practice [11, 12].

PD-L1 expression on tumor or immune cells has emerged as a potential predictive biomarker for sensitivity to ICIs and patient stratification[13]. According to National Comprehensive Cancer Network (NCCN) guidelines, pembrolizumab, pembrolizumab with chemotherapy, atezolizumab, and cemiplimab are the first-line immunotherapy regimens for advanced non-squamous NSCLC with PD-L1 expression ≥ 50%. These treatments have demonstrated better PFS and OS compared to platinum-based double chemotherapy[14–17]. However, direct comparisons of different PD-1/PD-L1 inhibitors, including sintilimab and tislelizumab, have not been performed as they have recently entered the market[18].

The aim of our study is to evaluate the effectiveness and ranking of ICIs in advanced NS-NSCLC with PD-L1 expression ≥ 50%. By doing so, our results may provide valuable insights into the most effective treatment for advanced NS-NSCLC with high PD-L1 expression.

## Materials and methods

**Fig. 1:**
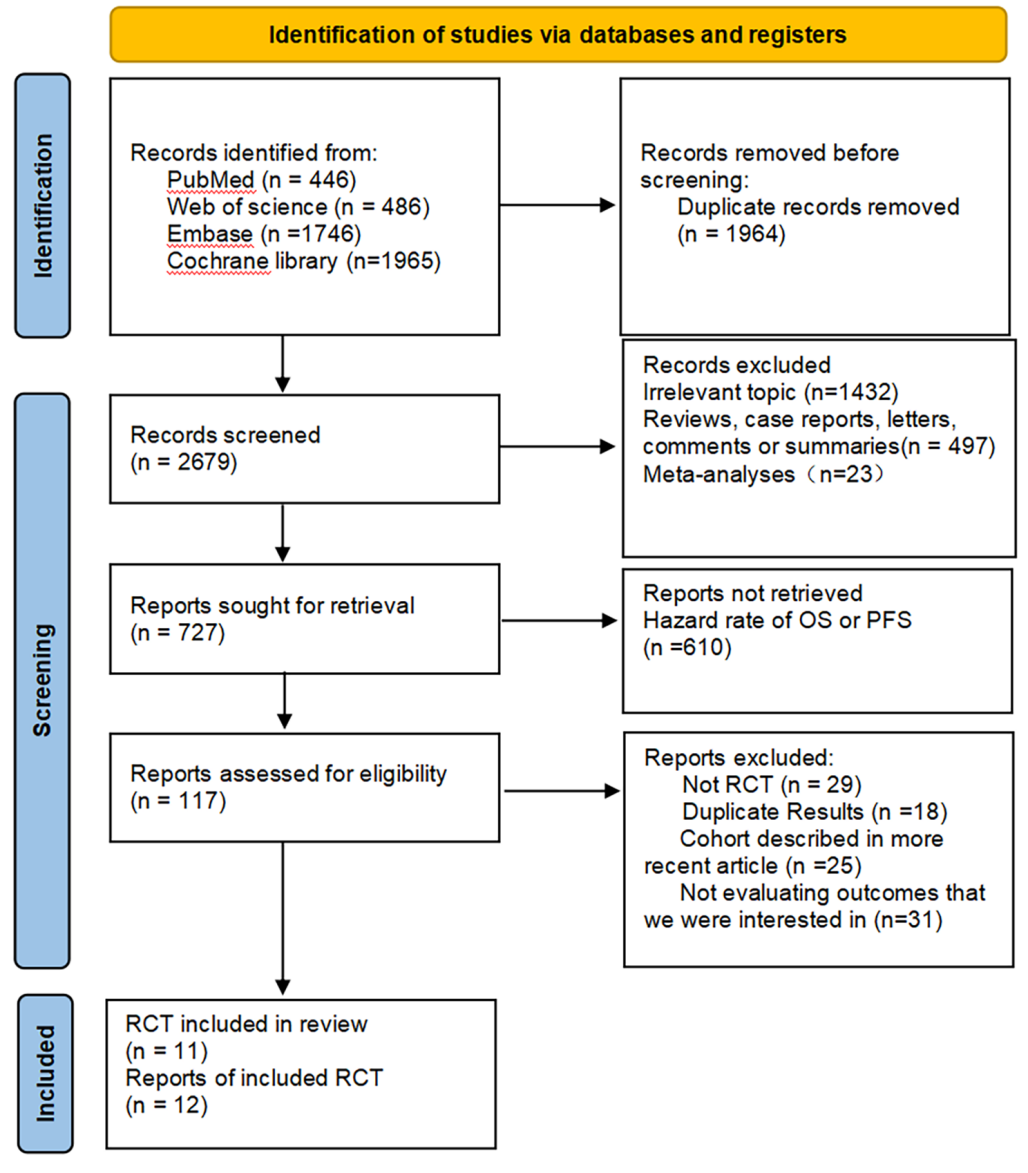
Flow diagram depicting the process of searching for and selecting relevant literature

The NMA in this study was conducted in accordance with the Preferred Reporting Items for Systematic Reviews and Meta-Analyses (PRISMA-NMA) extension statement, as shown in Fig. 1 [19]. Bayesian methods were employed to enable indirect comparisons between treatments that have not yet been directly compared in clinical trials, thus allowing for probabilistic predictions of treatment outcomes [20]. To ensure transparency, reliability, and originality, the protocol for this study was registered in the Prospective Register of Systematic Reviews (PROSPERO) under the reference number CRD42022349712.

### Data sources and search strategy

To identify relevant studies, a systematic search was conducted in the PubMed, Cochrane Library, Web of Science, and Embase databases from the date of database creation to October 15, 2022, using the following keywords: “non-small-cell lung cancer,“ “randomized clinical trial,“ “immunotherapy,“ “PD-1,“ “PD-L1,“ “CTLA-4,“ “pembrolizumab,“ “atezolizumab,“ “nivolumab,“ “ipilimumab,“ “durvalumab,“ “tislelizumab,“ “camrelizumab,“ “cemiplimab,“ and “sintilimab.“

### Selection criteria

For the inclusion criteria, we selected studies for this meta-analysis based on the following:

1. Phase II or Phase III RCTs were considered.
2. Patients with histologically or cytologically confirmed stage IV NSCLC were included in the RCT.
3. The RCT involved treatment with ICIs.
4. Availability of OS or PFS data was required for NS-NSCLC patients with PD-L1 expression ≥ 50%.

On the other hand, the following studies were excluded from this meta-analysis:

1. RCTs involving the same patient group were excluded.
2. Editorials, observational studies, and reviews were not included.

### Data extraction and quality assessment

Data Extraction: The selected studies were subjected to a rigorous data extraction process in accordance with PRISMA guidelines. To ensure accuracy and completeness, three researchers independently extracted relevant data, with any discrepancies being resolved through discussion with a fourth author. The extracted data included details such as trial name, first author, source of publication, year of publication, trial phase, national clinical trial identification number, sample size, patient age and gender distribution, trial group, and control group. Clinical outcomes such as hazard ratios (HRs) and 95% confidence intervals (CIs) for OS and PFS were also extracted.

Quality Assessment: To ensure that the included studies met high-quality standards, the Cochrane Risk of Bias tool (2.0) was used to assess the quality of the RCTs. This tool evaluated the risk of bias in five key domains, including the randomization process, potential deviation from the intended intervention, missing outcome data, outcome measurement, and selection of reported results[21].

Based on the quality assessment results, the included studies were categorized as low-risk, high-risk, or unclear. This ensured that only studies with rigorous and robust methodologies were included in the meta-analysis.

### Statistical analysis

The Bayesian framework was employed using R software (version 4.0.3) with the “JAGS” and “GeMTC” packages to conduct the NMA, which aimed to evaluate the effectiveness of various ICIs in treating advanced NSCLC[22, 23]. A fixed-effect consistency model was utilized, and 20,000 simultaneous iterations and 50,000 sample iterations per chain were run on three independent Markov chains. The NMA endpoints were OS and PFS, with effect sizes measured by HRs and corresponding 95% CIs. For the head-to-head meta-analysis, the Revman software (version 5.4) was used. The rank probability command was utilized to rank the treatments from best to worst, and statistical significance was determined at a bilateral alpha level of less than 0.05. One reviewer performed the statistical analysis, and the results were checked by three other reviewers to ensure accuracy.

### Sensitivity analysis

In addition, to ensure the best fit for our analyses, we conducted a model comparison using the Deviance Information Criterion (DIC), which assesses the relative goodness-of-fit of the fixed-effect and random-effect models. A smaller DIC value indicates a better model fit. If the difference in DIC between the fixed-effect and random-effect models was less than 5, it was considered that the models were consistent. This approach helped us ensure that the most appropriate model was selected for each analysis cohort[24].

### Heterogeneity analysis

We performed a heterogeneity analysis using the “anote” command to calculate I^2^ values. I^2^ values were interpreted as follows: if the I^2^ value was less than 25%, it was considered low heterogeneity; if it was between 25% and 50%, it was considered medium heterogeneity; and if it was greater than 75%, it was considered high heterogeneity. In cases of low heterogeneity, a fixed-effects model was used, while a random-effects model was used in cases of medium or high heterogeneity[25].

## Results

### Studies included in the NMA

After conducting a thorough search of four databases - namely PubMed, Web of Science, Embase, and the Cochrane Library - a total of 4643 articles were identified. Following the removal of duplicates, 2679 articles were excluded from the analysis. The final selection process is depicted in Fig. 1, resulting in the inclusion of 12 articles in this NSCLC NMA. This meta-analysis included 2,176 patients from 11 RCTs, which evaluated the following 12 treatment options for NSCLC: atezolizumab plus chemotherapy (atezo-chemo), atezolizumab plus bevacizumab plus chemotherapy (atezo-beva-chemo), pembrolizumab (pem), pembrolizumab plus ipilimumab (pem-ipi), pembrolizumab plus chemotherapy (pem-chemo), nivolumab plus bevacizumab plus chemotherapy (nivo-beva-chemo), camrelizumab plus chemotherapy (camre-chemo), sintilimab plus chemotherapy (sinti-chemo), tislelizumab plus chemotherapy (tisle-chemo), cemiplimab (cemi), chemotherapy (chemo), and bevacizumab plus chemotherapy (beva-chemo). The details of the RCTs are provided in Table 1.

**Table 1:** Baseline characteristics of the studies included in the network Meta-analysis

Study	Phase	Design	Year	Registered ID	Randomization	Sample Size	Intervention Arm	Control Arm	Line of treatment
CameL	III	open-label	2020	NCT03134872	1:1	30/20	Camrelizumab + Chemotherapy (Carboplatin AUC 5 + Pemetrexed 500 mg/m^2^ Q3W)	Chemotherapy (CarboplatinAUC5 + Pemetrexed 500 mg/m^2^ Q3W)	First-line
IMpower130	III	open-label	2019	NCT02367781	2:1	88/42	Atezolizumab 1200 mg Q3W + Chemotherapy (Carboplatin AUC6 Q3W + Nab-paclitaxel 100 mg/m^2^ QW))	Chemotherapy (CarboplatinAUC6 Q3W + Nab-paclitaxel 100 mg/m^2^ QW)	First-line
IMpower132	III	open-label	2020	NCT02657434	1:1	25/20	Atezolizumab 1200 mg Q3W + Chemotherapy (Carboplatin AUC 6 or Cisplatin 75 mg/m^2^ + Pemetrexed 500 mg/m^2^ Q3W)	Chemotherapy (Carboplatin AUC 6 or Cisplatin 75 mg/m^2^ + Pemetrexed 500 mg/m^2^ Q3W)	First-line
IMpower150	III	open-label	2020	NCT02366143	1:1:1	71/72/63	Arm 1: Atezolizumab 1200 mg Q3W + Chemotherapy (Carboplatin AUC 6 + Paclitaxel 200 mg/m^2^ Q3W) Arm 2: Atezolizumab 1200 mg Q3W + Bevacizumab 15 mg/kg Q3W + Chemotherapy (Carboplatin AUC 6 + Paclitaxel 200 mg/m^2^ Q3W)	Bevacizumab 15 mg/kg Q3W + Chemotherapy (Carboplatin AUC 6 + Pemetrexed 200 mg/m^2^ Q3W)	First-line
KEYNOTE-024	III	open-label	2016	NCT02142738	1:1	125/124	Pembrolizumab 200 mg Q3W	Chemotherapy (Platinum-based Chemotherapy Regimens)	First-line
KEYNOTE-189	III	double-blind	2020	NCT02578680	2:1	132/70	Pembrolizumab 200 mg Q3W + Chemotherapy (Carboplatin AUC 5 or Cisplatin 75 mg/m^2^ + Pemetrexed 500 mg/m^2^ Q3W)	Chemotherapy (Carboplatin AUC 5 or Cisplatin 75 mg/m^2^ + Pemetrexed 500 mg/m^2^ Q3W)	First-line
KEYNOTE-598	III	double-blind	2020	NCT03302234	1:1	207/203	Ipilimumab 1 mg/kg Q6W + Pembrolizumab 200 mg Q3W	Pembrolizumab 200 mg Q3W	First-line
ORIENT-11	III	double-blind	2020	NCT03607539	2:1	107/61	Sintilimab200mg + Chemotherapy (Carboplatin AUC 5 or Cisplatin 75 mg/m^2^ + Pemetrexed 500 mg/m^2^ Q3W)	Chemotherapy (Carboplatin AUC 5 or Cisplatin 75 mg/m^2^ + Pemetrexed 500 mg/m^2^ Q3W)	First-line
RATIONALE 304	III	open-label	2020	NCT03663205	2:1	74/36	Tislelizumab 200 mg + Chemotherapy (Carboplatin AUC 5 or Cisplatin 75 mg/m^2^ + Pemetrexed 500 mg/m^2^ Q3W)	Chemotherapy (Carboplatin AUC 5 or Cisplatin 75 mg/m^2^ + Pemetrexed 500 mg/m^2^ Q3W)	First-line
EMPOWER-Lung 1	III	open-label	2021	NCT03088540	1:1	161/159	Cemiplimab 350 mg Q3W	Platinum-doublet Chemotherapy	First-line
TASUKI-52	III	double-blind	2021	NCT03117049	1:1	73/74	Nivolumab 360 mg Q3W + Bevacizumab 15 mg/kg Q3W + Chemotherapy(Carboplatin at AUC 6, paclitaxel 200 mg/m^2^)	Bevacizumab 15 mg/kg Q3W + Chemotherapy(Carboplatin at AUC 6, paclitaxel 200 mg/m^2^)	First-line

### Characteristics of studies

**Fig. 2:**
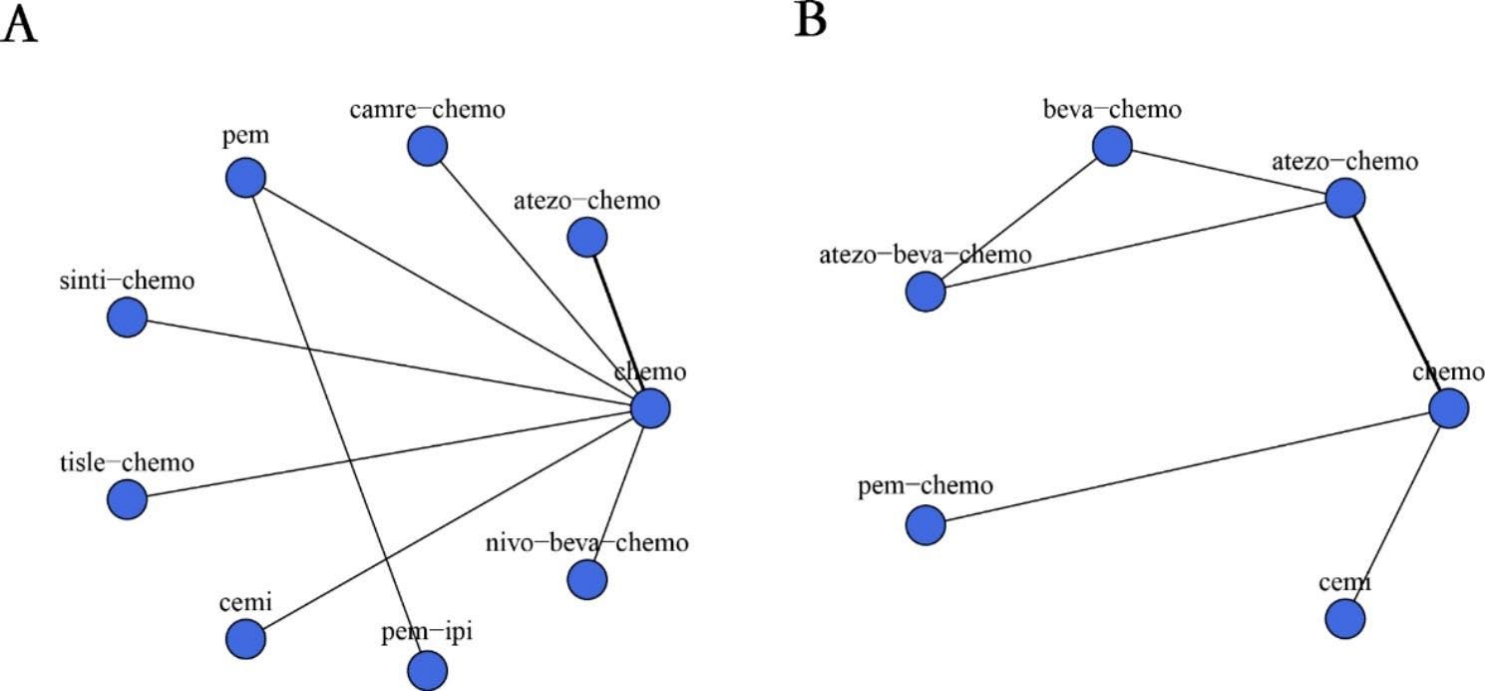
Network plot for effectiveness of 9 and 8 different treatment modalities for patients with PD-L1 expression⩾50% for PFS (**A**) and OS (**B**), Respectively. Circles represent the intervention as a node in the network; lines represent direct comparisons within the frame of RCTs; the line thickness indicates the number of RCTs included in each comparison. Atezo, atezolizumab; Beva, bevacizumab; Camre, camrelizumab; Cemi, cemiplimab; Chemo, chemotherapy; Pem, pembrolizumab; Sinti, sintilimab; Tisle, Tislelizumab; Nivo, nivolumab

The experimental group of 3 RCTs including ICI monotherapy(KN-024[17], EMPOWER-Lung1[15]), and experimental groups in eight trials studied ICIs in combination with chemotherapy (Camel[26], IMpower130[27], IMpower132[16, 28], IMpower150[29], KN-189[30], ORIENT-11[31], RATIONALE304[32], TASUKI-52[33]). In addition, an RCT evaluated the combination of PD-1 inhibitors with CTLA-4 inhibitors(Pembrolizumab/ Ipilimumab.KN-598[34]). Figure 2 displays a network plot of the eligible comparisons.

### Assessment of included trials

**Fig. 3:**
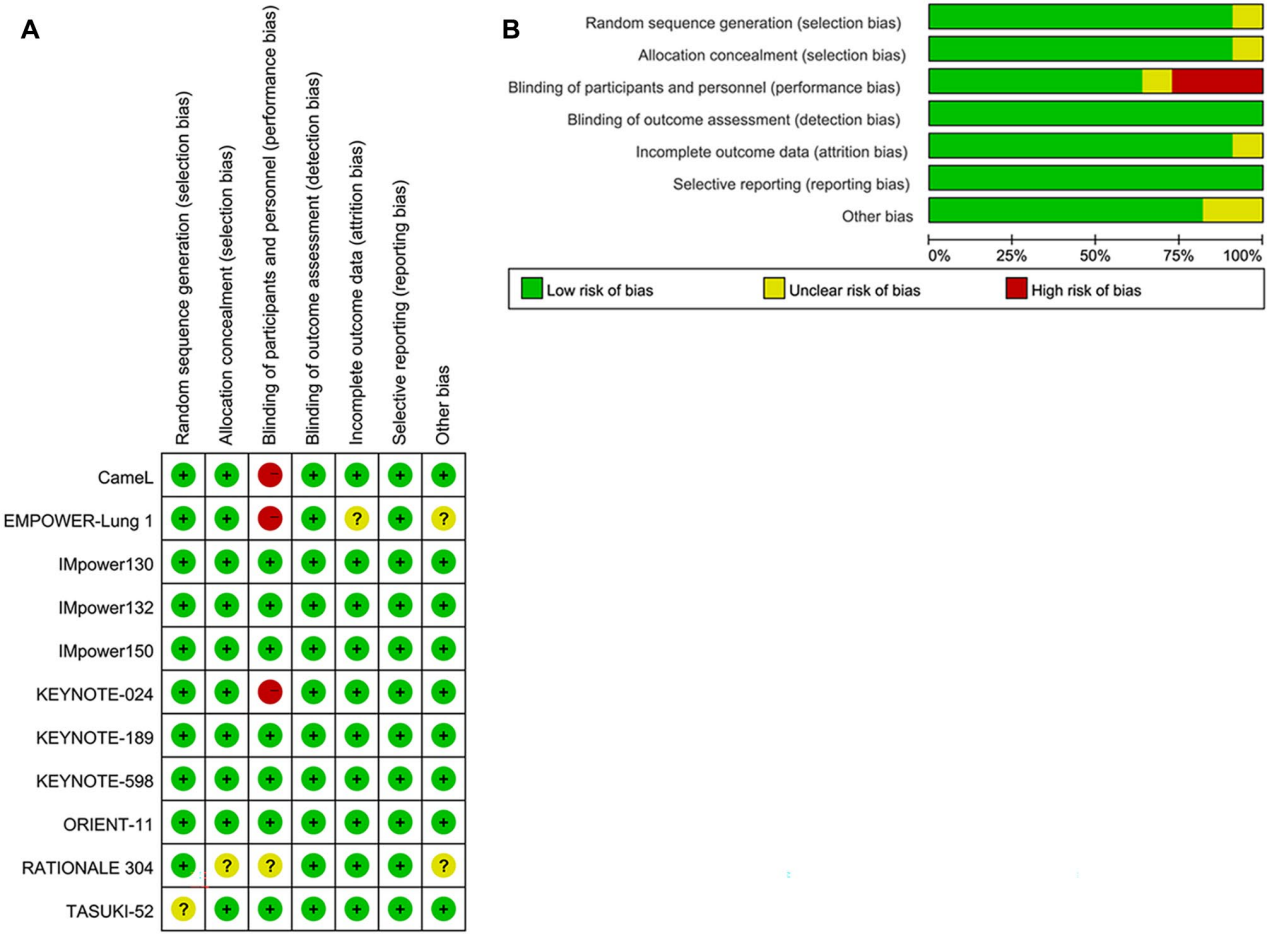
Risk of Bias Figure. (**A**) methodological quality summary: authors’ judgment about each methodological quality item for each included study. Performance bias and detection bias presented were for risk of bias; (**B**) Methodological quality graph: authors’ judgment about each methodological quality item presented as percentages across all included studies

Figure 3 presents the results of the risk of bias assessment for the 11 included trials. Overall, the risk of bias was low as all studies were well-designed randomized controlled trials. Trial protocols were accessed to confirm methodological information. In the selection bias domain, 10 trials were rated as low risk, while one trial (TASUKI-52) was rated as unclear. For the reporting bias domain, 10 trials were rated as low risk, while one trial (RATIONALE 304) was rated as unclear. In the performance bias domain, seven trials were rated as low risk, three trials (Camel, EMPOWER-Lung 1, KN-024) were rated as high risk, and one trial (RATIONALE 304) was rated as unclear. As for the detection bias domain, all trials were rated as low risk, given that lack of blinding is unlikely to affect this domain. In the attrition bias domain, 10 trials were rated as low risk, and one trial (EMPOWER-Lung 1) was rated as unclear. For the reporting bias domain, all trials were rated as low risk, mainly because they were analyzed based on the intention-to-treat population and reported sufficient endpoints. However, some trials allowed crossover, which was deemed a potential source of bias.

### Pairwise meta-analysis

Paired meta-analyses were performed for four trials that reported HRs for OS and seven trials that reported HRs for PFS when comparing ICIs to chemotherapy.

The head-to-head comparisons revealed that, in comparison to chemotherapy, patients treated with atezolizumab plus chemotherapy had improved OS (HR, 0.81; 95% CI, 0.52–1.26) and improved PFS (HR, 0.50; 95% CI, 0.35–0.71). Additionally, patients treated with pembrolizumab had improved OS (HR, 0.59; 95% CI, 0.40–0.87) and improved PFS (HR, 0.55; 95% CI, 0.39–0.77). Patients treated with cemiplimab had improved OS (HR, 0.64; 95% CI, 0.43–0.94) and improved PFS (HR, 0.60; 95% CI, 0.44–0.82). Moreover, patients treated with camrelizumab plus chemotherapy (HR, 0.39; 95% CI, 0.15–1.04), sintilimab plus chemotherapy (HR, 0.31; 95% CI, 0.20–0.49), and tislelizumab plus chemotherapy (HR, 0.31; 95% CI, 0.17–0.56) had improved PFS.

**Fig. 4:**
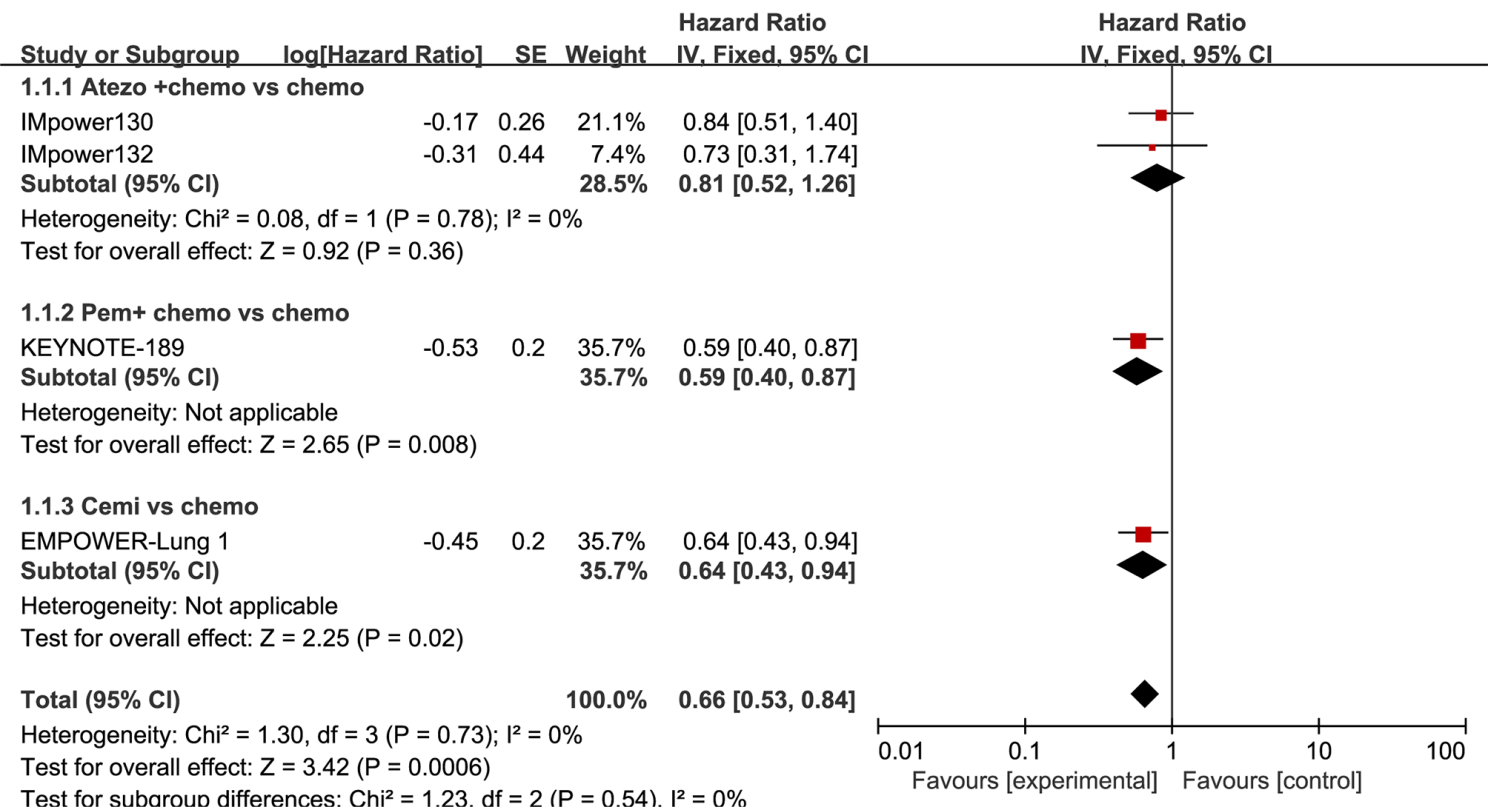
Forest plot for OS. The efficacy of ICIs vs. chemotherapy for Advanced NSCLC in Patients with PD-L1 Expression ⩾50%

**Fig. 5:**
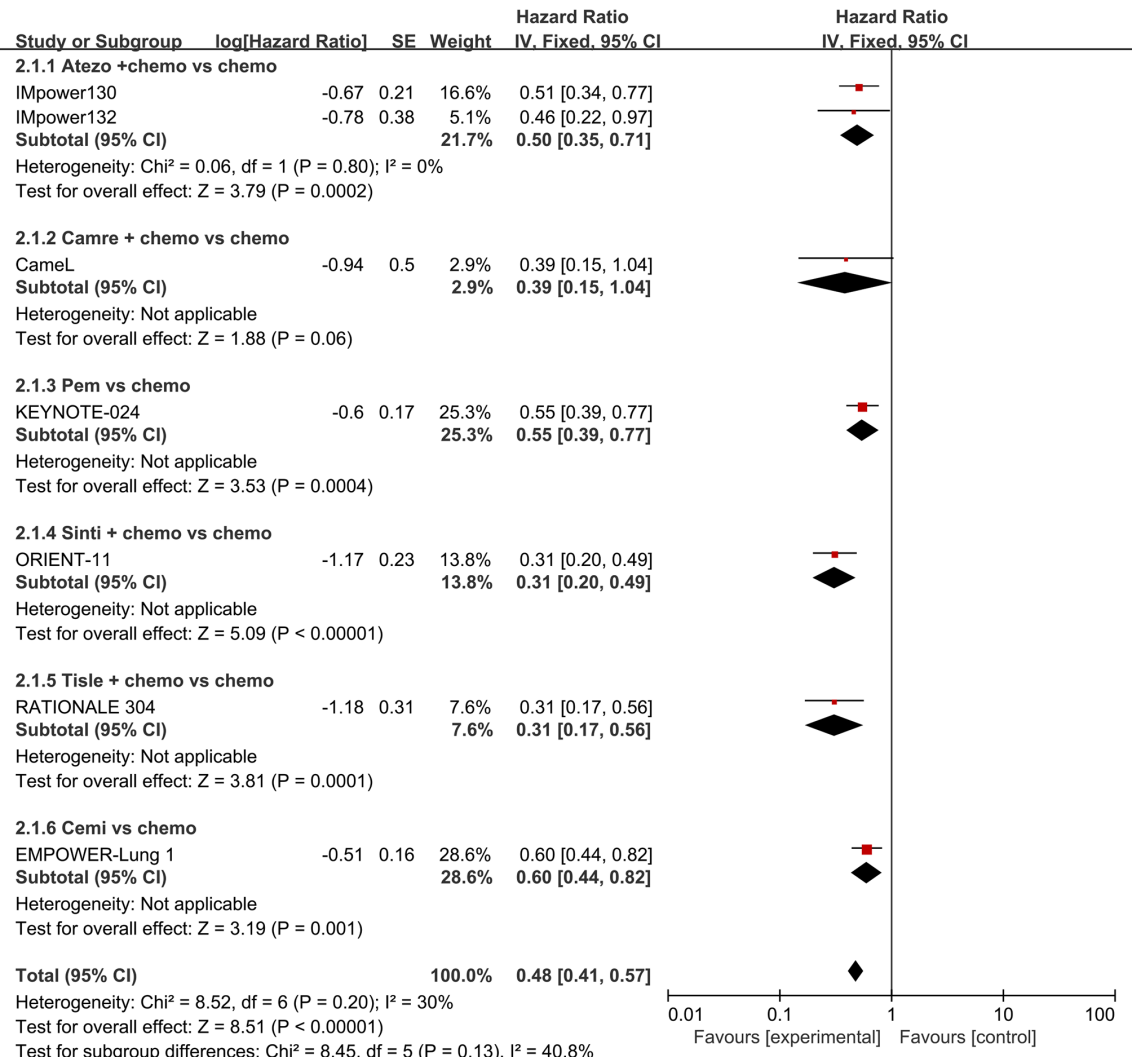
Forest plot for PFS. The efficacy of ICIs vs. chemotherapy for Advanced NSCLC in Patients with PD-L1 Expression ⩾50%

The forest plots used to compare the pairwise results of OS and PFS are displayed in Figs. 4 and 5, respectively.

### Network Meta-Analysis

**Fig. 6:**
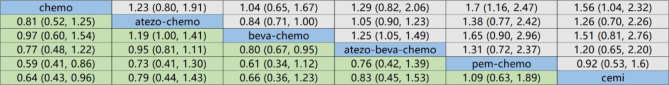
Summary for target outcomes including OS. Efficacy profiles of the Bayesian network meta-analysis in patients with advanced NSCLC in Patients with PD-L1 Expression ⩾50%

Regarding OS, the indirect comparison results are shown in Fig. 6, compared to chemotherapy, atezolizumab plus bevacizumab plus chemotherapy (HR, 0.77; 95%CI, 0.48–1.22).

**Fig. 7:**
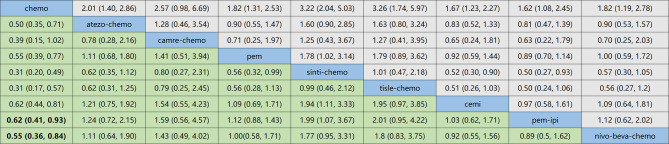
Summary for target outcomes including PFS. Efficacy profiles of the Bayesian network meta-analysis in patients with advanced NSCLC in Patients with PD-L1 Expression ⩾50%

Regarding PFS, the results of the indirect comparison are shown in Fig. 7, compared to chemotherapy, pembrolizumab plus ipilimumab (HR, 0.62; 95%CI, 0.41–0.93), nivolumab plus bevacizumab plus chemotherapy (HR, 0.55; 95%CI, 0.41–0.93).

### Rankings

**Fig. 8:**
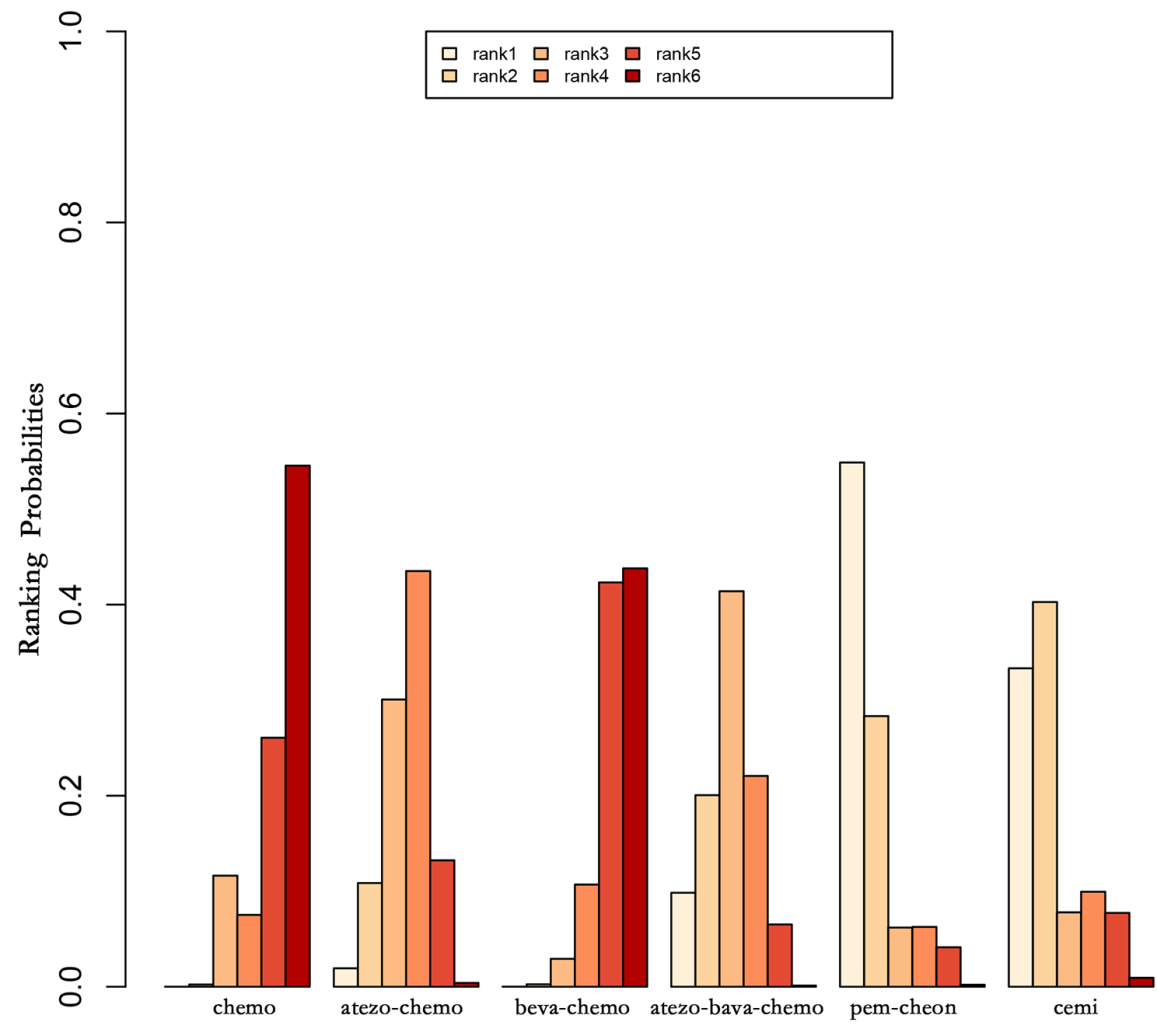
Ranking of overall survival (OS)

**Fig. 9:**
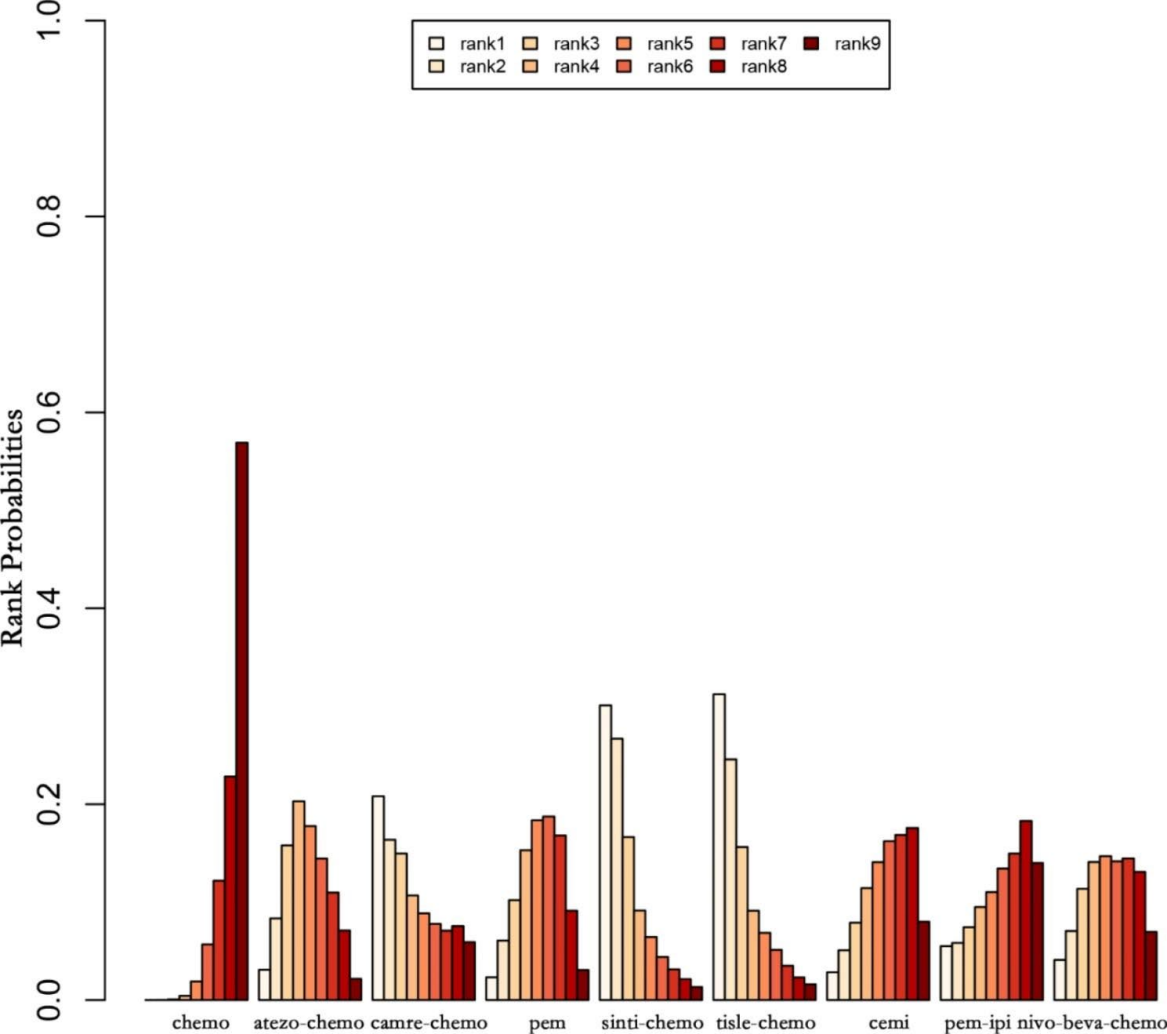
Ranking of Progression-Free-Survival (PFS)

Figures 8 and 9 present the summary of treatment ranking probabilities for the comparative efficacy of OS and PFS, respectively.

For OS, pembrolizumab plus chemotherapy had the highest probability of being the most effective treatment (54.87%), followed by cemiplimab (40.27%) and atezolizumab plus bevacizumab plus chemotherapy (41.4%) in second and third place, respectively.

For PFS, tislelizumab plus chemotherapy was ranked first with a probability of 31.22%, followed by sintilimab plus chemotherapy in second place with a probability of 26.67%, and atezolizumab plus chemotherapy in third place with a probability of 15.78%.

## Discussion

This groundbreaking study represents the most comprehensive NMA to date, providing an in-depth analysis of the efficacy of first-line immunotherapy in patients with NS-NSCLC and PD-L1 expression levels ≥ 50%. Notably, this study stands out from other NMAs in its inclusion of RCTs utilizing sintilimab and tislelizumab for treatment. The study’s analysis involved an impressive cohort of 2037 advanced non-squamous NSCLC patients with PD-L1 expression ≥ 50%, culled from 12 RCTs. The results showed that compared to chemotherapy alone, the use of chemotherapy-ICI, monotherapy ICI, and ICI-ICI regimens resulted in higher PFS and OS rates. Squamous NSCLC is a particularly complex disease, impacted by multiple factors, predominantly linked to smoking, which results in a high mutation rate in its genes[35]. Non-Small Cell Lung Adenocarcinoma is more common in non-smokers or light smokers and tends to occur in younger individuals[36]. It is more likely to develop in the outer regions of the lung.Non-Small Cell Lung Adenocarcinoma can present as a solitary nodule, multiple nodules, or as a diffuse infiltrative pattern. Compared to squamous NSCLC, non-squamous NSCLC is characterized by simpler mutations, which offers insight into why non-squamous NSCLC patients with PD-L1 expression ≥ 50% can benefit from various ICI treatment regimens[37]. These findings hold immense promise for improving the treatment of advanced NS-NSCLC patients with PD-L1 expression ≥ 50%, and bring us one step closer to more effective, personalized therapies for lung cancer[38].

The NMA is an expansion on the traditional meta-analysis, which indirectly compares interventions in RCTs through a common comparison group, in addition to the support of multitude of studies to ensure the validity of the results. To assess the relative effectiveness of chemotherapy-ICI and ICI-ICI in treating advanced non-squamous NSCLC with PD-L1 expression ≥ 50%, head-to-head clinical trials represent the most informative approach, providing valuable insights for clinical decision-making.

Despite the promising results of immune checkpoint inhibitors for the treatment of NSCLC, it is evident that certain patients may not respond optimally to these therapies. As such, the identification of predictive biomarkers has emerged as a critical strategy to guide the personalized selection of immunotherapy, ensuring that patients receive the most effective treatment available. These biomarkers provide valuable insights into the unique molecular characteristics of individual patient’s tumors, enabling oncologists to make informed decisions regarding the use of immune checkpoint inhibitors in the management of NSCLC[39]. PD-L1 has been identified as a good predictive biomarker and NSCLC patients with high PD-L1 expression tend to respond better to the use of immune checkpoint inhibitors [40, 41]. After conducting a comprehensive analysis of seven RCTs, we have determined that pembrolizumab in combination with chemotherapy outperforms all other included therapeutic agents in terms of OS benefit. In fact, the findings of the Dafni et al. meta-analysis support the superiority of pembrolizumab, particularly in the management of NSCLC patients with a PD-L1 expression level of 50% or higher[42]. This is consistent with our findings, suggesting that the beneficial immunotherapy approach for NSCLC and NS-NSCLC with PD-L1 expression ≥ 50% does not differ significantly. Our comprehensive analysis of nine RCTs has revealed that tislelizumab in combination with chemotherapy and sintilimab in combination with chemotherapy offers superior PFS benefits compared to chemotherapy and all other included therapeutic agents. Interestingly, our analysis also revealed no significant difference in PFS between tisle-chemo and sinti-chemo. Furthermore, our findings indicate that PD-1 inhibitors in combination with chemotherapy are more efficacious than PD-L1 inhibitors in combination with chemotherapy. This observation can be attributed to the fact that PD-1 antibodies can block the binding of PD-1 to both PD-L1 and PD-L2, thus more fully inhibiting the occurrence of immune escape, leading to better treatment outcomes for patients with advanced cancers, including non-small cell lung cancer[43].

While our study provides valuable insights into the efficacy of chemotherapy and immune checkpoint inhibitors (ICI) in the management of cancer, there are still some limitations that should be considered. Firstly, the included trials used different methods to detect PD-L1 expression cut-offs, and in several trials, the investigators did not specify the method used to detect PD-L1 expression cut-offs. This inconsistency in measurement could lead to some unintentional misclassification, resulting in an underestimation or overestimation of the benefits of chemotherapy and ICI. For example, the SP142 method, which is used to measure PD-L1 expression in tumor cells, is less sensitive than other methods, potentially impacting the accuracy of our findings[44]. Secondly, four of the included studies reported reporting both OS and PFS, with the remaining eight included studies reporting only OS or PFS. As a result, it was impossible to conduct direct comparisons between many treatment modalities, leading to some limitations in our ability to draw definitive conclusions. Thirdly, it’s important to acknowledge that there is no universally accepted metric for assessing the efficacy of immune checkpoint inhibitor (ICI) treatments for lung cancer. While we used overall survival and progression-free survival as the primary endpoints in our study, it’s important to note that they do not capture the full range of treatment benefits that may be experienced by patients. Other important metrics, such as health-related quality of life, should also be considered when evaluating the efficacy of ICI treatments. It’s worth emphasizing that health-related quality of life is an especially valuable metric for patients undergoing ICI treatment for lung cancer. Beyond the clinical endpoints of overall survival and progression-free survival, patients often prioritize their physical and emotional well-being. As such, assessing treatment efficacy through patient-reported outcomes that capture the quality of life can provide a more comprehensive understanding of the benefits and limitations of ICI treatments in real-world settings. Fourth, we did not evaluate the safety of the ICIs due to the lack of safety data for PD-L1 expression ≥ 50% in advanced NS-NSCLC reported in the included studies. Lastly, it’s important to acknowledge that the results of our network meta-analysis (NMA) should be interpreted with caution due to the limited number of randomized controlled trials and participants included in our study. While our analysis provides valuable insights into the relative efficacy and safety of the various immune checkpoint inhibitor therapies for non-small cell lung cancer, it’s essential to note that further research is needed to confirm and expand upon our findings. Given the complexity and heterogeneity of lung cancer, it’s crucial to approach treatment selection on an individualized basis. In this regard, targeted histological staging and stratification of PD-L1 expression may be key factors to consider when selecting an appropriate immune checkpoint inhibitor therapy for a given patient. By tailoring treatment based on individual patient characteristics, we can optimize treatment outcomes and improve overall survival rates in this patient population.

In light of these considerations, we recommend that clinicians and researchers continue to explore the differences in efficacy and safety of immune checkpoint inhibitor therapies for non-small cell lung cancer through well-designed, rigorous studies that take into account the full range of patient factors and clinical outcomes. Only through such efforts can we achieve truly personalized and effective treatments for this devastating disease.

## Conclusions

In conclusion, this NMA demonstrated that for NS-NSCLC with PD-L1 ≥ 50%, pembrolizumab plus chemotherapy, tislelizumab plus chemotherapy and sintilimab plus chemotherapy appear to be good treatment options. For this group of patients, ICI alone, ICI in combination with chemotherapy drugs, or a combination of two ICIs is more effective than chemotherapy drugs.

## Data Availability

All data generated or analysed during this study are included in this published article.

## Abbreviations

NSCLC: Non-small Cell Lung Cancer
NS-NSCLC: Non-squamous Non-small Cell Lung Cancer
PD-1: Programmed Cell Death-1
PD-L1: Programmed Cell Death 1 Ligand 1
ICIs: Immune Checkpoint Inhibitors
NMA: Network Meta-analysis
RCT: Randomized Controlled Trials
OS: Overall Survival
PFS: Progression-free Survival
CTLA-4: Cytotoxic T Lymphocyte-Associated Antigen-4
MCMC: Markov chain Monte Carlo
